# Low‐dose naltrexone and opioid consumption: a drug utilization cohort study based on data from the Norwegian prescription database

**DOI:** 10.1002/pds.4201

**Published:** 2017-03-29

**Authors:** Guttorm Raknes, Lars Småbrekke

**Affiliations:** ^1^ Regional Medicines Information and Pharmacovigilance Centre (RELIS)University Hospital of North NorwayTromsøNorway; ^2^ National Centre for Emergency Primary Health CareUni Research HealthBergenNorway; ^3^ Department of Pharmacy, Faculty of Health SciencesUiT—The Arctic University of NorwayTromsøNorway

**Keywords:** naltrexone, opioids, painkillers, drug utilization, prescription database, off label prescribing, pharmacoepidemiology

## Abstract

**Purpose:**

Low‐dose naltrexone (LDN) is used in a wide range of conditions, including chronic pain and fibromyalgia. Because of the opioid antagonism of naltrexone, LDN users are probably often warned against concomitant use with opioids. In this study, based on data from the Norwegian prescription database, we examine changes in opioid consumption after starting LDN therapy.

**Methods:**

We included all Norwegian patients (*N* = 3775) with at least one recorded LDN prescription in 2013 and at least one dispensed opioid prescription during the 365 days preceding the first LDN prescription. We allocated the patients into three subgroups depending on the number of collected LDN prescriptions and recorded the number of defined daily doses (DDDs) on collected prescriptions on opioids, nonsteroidal anti‐inflammatory drugs and other analgesics and antipyretics from the same patients.

**Results:**

Among the patients collecting ≥4 LDN prescriptions, annual average opioid consumption was reduced by 41 DDDs per person (46%) compared with that of the previous year. The reduction was 12 DDDs per person (15%) among users collecting two to three prescriptions and no change among those collecting only one LDN prescription. We observed no increase in the number of DDDs in nonsteroidal anti‐inflammatory drugs or other analgesics and antipyretics corresponding to the decrease in opioid use.

**Conclusions:**

Possibly, LDN users avoided opioids because of warnings on concomitant use or the patients continuing on LDN were less opioid dependent than those terminating LDN. Therapeutic effects of LDN contributing to lower opioid consumption cannot be ruled out. © 2017 The Authors. Pharmacoepidemiology & Drug Safety Published by John Wiley & Sons Ltd.

## Introduction

Naltrexone is an opioid antagonist that was approved by the FDA as treatment of heroin addiction in 1984 and against alcohol dependence in 1995.^1^ Over the past 20 years, some doctors and patients have claimed that low–dose naltrexone (LDN, 5 mg/day or less) may have beneficial therapeutic effect in autoimmune diseases like multiple sclerosis and inflammatory bowel disease,^2^ and there have been some promising preliminary studies.^3^, ^4^ LDN has also gained popularity among patients with chronic pain,^5^, ^6^ and two small studies study indicate beneficial effects in patients with fibromyalgia.^7^, ^8^ By June 2016, LDN was rated by patients as the second best of 138 fibromyalgia treatments in CureTogether, a large patient Internet forum.^9^ Alcohol and opioid dependence are the only approved indications for naltrexone, and other use is off label.

On 27 February 2013, the biggest commercial TV channel station in Norway (TV2) aired a documentary on the alleged effects of LDN. Patients with severe multiple sclerosis claimed that the use of LDN almost normalized their function.^10^ The documentary led to an immediate and large increase in the consumption of LDN among patients with a wide range of diagnoses. According to the Norwegian Prescription Database (NorPD), the number of naltrexone users rose from less than 20 in 2012 to more than 15000 in 2013 and 2014.^11^

There was also significant interest among Norwegian chronic pain and fibromyalgia patients, and the Norwegian Fibromyalgia association issued a statement in which they justified testing LDN.^12^ There are no scientific data available, but it is likely that a fair number of patients with chronic pain conditions tried this treatment, encouraged by the LDN documentary.

Concomitant use of opioids and naltrexone is often discouraged. Theoretically, the opioid antagonism by naltrexone, even in low doses, may reduce the opioid effect or induce withdrawal syndromes in opioid‐dependent individuals.^13^ It is hypothesized that endogenous opioids may interfere with the alleged beneficial effects of LDN.^14^ LDN Internet resources, including Norway's biggest LDN forum and some professionals who have experience in LDN therapy, generally warn against opioid–naltrexone combinations.^15^

The main objective of this study is to examine whether initiation of LDN therapy was associated with change in opioid consumption. Our main hypothesis was that patients used less opioids after starting LDN. The secondary objective was to investigate whether starting LDN was associated with change in collection of prescriptions of other painkillers like nonsteroidal anti‐inflammatory drugs (NSAIDs) or other analgesics and antipyretics, in addition to time to first opioid prescription. Our secondary hypothesis was that a reduction in defined daily doses (DDDs) in collected prescriptions of opioids would be associated with an increase in number of DDDs of NSAIDs and other analgesics and antipyretics.

## Methods

### Setting

This study used data from the NorPD that contain individual data on all prescriptions dispensed since 2004 to the entire Norwegian population living outside hospitals. Details on NorPD are published elsewhere.^16^ In short, for each prescription, the NorPD contains a unique pseudonym for the personal identifier and demographic data on patient and prescriber, the specialty of the prescriber, the Anatomical Therapeutic Chemical classification (ATC) code and the amount of drug in DDDs, date of dispensing and location of the dispensing pharmacy. It is possible to follow prescriptions to individual patients on both reimbursed and non‐reimbursed prescriptions. Only prescriptions on products that have a product identifying number are recorded, and pharmacy produced specialty products are not included. The database contains no information on the indication for therapy but has disease codes (ICD‐10 or ICPC‐2) for reimbursed medications. The Norwegian Institute of Public Health is the host of the database.^17^ We used the following variables in this study: person identifier for patient, patient age and sex, ATC code, product identifying number, date of dispensing and dispensed volume in DDDs. For a fee, NorPD provided a data file according to their data access procedures.

### Low–dose naltrexone product and patients

All Norwegian patients with at least one LDN prescription recorded in NorPD in 2013 and at least one dispensed opioid prescription the preceding 365 days before the first LDN prescription according to NorPD were included in the study. For the included patients, we recorded all dispensed prescriptions of opioids, NSAIDs and other analgesics and antipyretics using ATC codes from the day of first LDN prescription +364 days.

Only one LDN product (Naltrekson Kragerø 3‐mg tab; Kragerø tablettproduksjon AS, Kragerø, Norway) was recorded in NorPD. This product was assigned a product identifying number (361181) on 15 May 2013, and in practice, there were no LDN prescriptions in NorPD before this date.

### Outcome variables

Main outcome was the difference between the cumulative collected amounts of DDDs on opioids during the 365 days preceding the first LDN prescription (index date) to the first year (day of first LDN prescription +364 days) following the first LDN prescription. We expressed the difference for each patient by subtracting the number of collected opioid DDDs in the year following the first LDN prescription from the number of DDDs in the year preceding the first LDN prescription.

As a secondary outcome, we used equivalent DDD changes in the collection of other analgesics and antipyretics (ATC N02B) and NSAIDs (ATC M01A, excluding glucosamine) and combined DDD change of opioids, other analgesics and antipyretics and NSAIDs. In contrast to opioids, some NSAIDs and other analgesics and antipyretics are available over‐the‐counter (OTC) in Norway. Consequently, the NorPD only partially captures the consumption of other analgesics and antipyretics and NSAIDs.

Time (days) to collection of first opioid prescription after the first LDN prescription was another secondary outcome.

We have previously documented a median daily LDN dose of 3.7 mg among patients collecting more than one LDN prescription (equivalent to 4.5 boxes of 100 tablets LDN per year) (9). We divided the patients into subgroups depending on the number of collected LDN prescriptions the first year after the index date. Patients were included in the persistent user (LDN × ≥4) subgroup if they collected four or more LDN prescriptions. The other subgroups were one‐time users (LDN × 1) and intermediate LDN users (LDN × 2–3).

### Statistical methods

Data were processed in Microsoft Excel 2010 and SPSS 23. Paired samples *t*‐tests were performed to compare total dispensed DDDs of opioids, other analgesics and antipyretics and NSAID 1 year before and 1 year after the first LDN prescription. All statistical tests were two‐tailed, and the alpha level was set to 0.05. Mean paired differences with 95% confidence intervals for differences were calculated. We also analyzed the cumulative opioid consumption per patient throughout the study period. We used linear regression to demonstrate the relationship between time before the index date and cumulative dose opioid and total dose painkillers for the LDN × ≥4 group. Extrapolation of these curves was used in diagrams to visualize any changes in consumption after the index date.

## Results

### Patients

Among the 11275 patients who collected at least one LDN prescription in 2013, 3775 collected at least one opioid prescription during the 365 days preceding their first LDN prescription. These patients collected 10 337 LDN prescriptions that were captured by NorPD within the study period. There were 36615 opioid prescriptions, 14391 NSAID prescriptions and 13783 prescriptions on other analgesics and antipyretics included in the study. In the first year following the first LDN prescription, 1347 of the included patients collected LDN once, 1152 two or three times and 1276 collected four or more LDN prescriptions.

**Figure 1:**
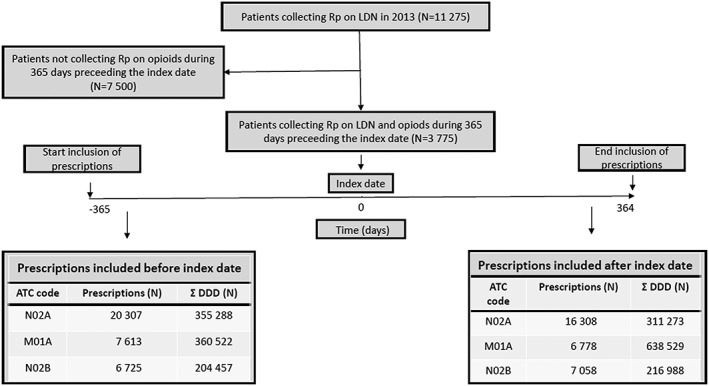
Flowchart showing the inclusion of patients and painkiller prescriptions from NorPD

The flow diagram in Figure 1 shows the inclusion of patients and prescriptions in the three painkiller groups.

### Descriptive data

Table 1 displays baseline data of the three subgroups. Age and gender distributions were the same in one‐time and persistent users. Patients in the LDN × 2–3 group had 1.9 years lower mean age and approximately one percentage point lower proportion of females. There were no missing data on age or gender among the included. Patients in the LDN × 1 group had collected opioids more frequently than the other subgroups before the first LDN prescription, and the LDN ≥4 group had higher mean combined number of dispensed opioid, other analgesic and antipyretic and NSAID prescriptions.

**Table 1:** Baseline data of the three subgroups

		LDN × 1	LDN × 2–3	LDN ≥4
*N*		1347	1152	1276
Mean age		53.2	51.5	53.2
Females (%)		78.5	77.8	78.6
Average number of prescriptions 1 year before first LDN prescription				
Opioid		3.9	3.1	3.3
Other analgesics and antipyretics		3.1	3.3	3.5
NSAID		2.1	2.4	2.6

For all included patients, 2‐year observation time was achieved, 1 year prior to and 1 year following the index date (7550 patient‐years in total).

### Outcome data

Mean number of dispensed opioid DDDs before and after the first LDN prescription is summarized in Table 2. The total amount of dispensed opioids was lower in the year following the first LDN prescription compared with the preceding year. The magnitude and statistical significance of the difference were increasing with the number of LDN prescriptions collected. In the LDN ≥4 group, average opioid reduction was 41 DDDs (46%) per person. The LDN × 1 group had a non‐significant reduction in opioid use, whereas the LDN × 2–3 group reduced opioids by 15%.

**Table 2:** Number of LDN dispenses and average amount (DDD) of opioids (ATC N02A) dispensed 1 year before and 1 year after the first LDN prescription in 2013 in Norway. Mean paired differences with 95% confidence intervals (CI), and *p*‐value for paired *t*‐test

Number of LDN dispenses (subgroup)	*N*	Opioids before	Opioids after	Mean paired difference		95% CI of difference (DDD)	*p*
		(DDD)	(DDD)	(DDD)	(%)		
LDN × 1	1347	112.3	107.3	−5.0	−4.4	−11.3 to 1.4	0.123
LDN × 2–3	1152	79.5	67.4	−12.1	−15.2	−17.1 to −7.0	<0.001
LDN ≥ 4	1276	90.1	49.0	−41.1	−45.7	−47.1 to −35.1	<0.001
Any LDN	3775	94.8	75.4	−19.4	−20.4	−22.8 to −15.9	<0.001

There was a minor difference in the use of other analgesics and antipyretics before and after the first prescription of LDN (Table 3). For all subgroups combined, there was a borderline significant mean increase of 2.7 DDDs (4.8%) (95%CI 0.1 to 5.3). The observed increase in each of the three subgroups did not reach statistical significance.

**Table 3:** Number of LDN dispenses and average amount (DDD) of other analgesics and antipyretics (ATC N02B) dispensed 1 year before and 1 year after the first LDN prescription in 2013 in Norway. Mean paired differences with 95% confidence intervals (CI), and *p*‐value for paired *t*‐test

Number of LDN dispenses (subgroup)	*N*	Other analgesics and antipyretics before	Other analgesics and antipyretics after	Mean paired difference		95% CI of difference (DDD)	*p*
		(DDD)	(DDD)	(DDD)	(%)		
LDN × 1	1347	48.4	51.4	3.0	6.3	−0.2 to 6.3	0.068
LDN × 2–3	1152	53.8	55.2	1.4	2.5	−4.7 to 7.5	0.660
LDN ≥4	1276	63.2	66.6	3.4	5.4	−0.7 to 7.6	0.107
Any LDN	3775	55.0	57.7	2.7	4.8	0.1 to 5.3	0.046

As shown in Table 4, there was a reduction in total amount of dispensed NSAID in all subgroups, but least pronounced in the LDN ≥4 group.

**Table 4:** Number of LDN dispenses and average amount (DDD) of NSAIDs (ATC M01A) dispensed 1 year before and 1 year after the first LDN prescription in 2013 in Norway. Mean paired differences with 95% confidence intervals (CI), and *p*‐value for paired *t*‐test

Number of LDN dispenses (subgroup)	*N*	NSAID before	NSAID after	Mean paired difference		95% CI of difference (DDD)	*p*
		(DDD)	(DDD)	(DDD)	(%)		
LDN × 1	1347	82.3	74.9	−7.4	(−9.0)	−13.6 to −1.2	0.020
LDN × 2–3	1152	94.6	85.5	−9.0	(−9.6)	−16.9 to −1.2	0.023
LDN ≥ 4	1276	111.4	104.0	−7.4	(−6.6)	−14.6 to −0.2	0.045
Any LDN	3775	95.9	88.0	−7.9	(−8.2)	−12.0 to −3.8	<0.001

**Figure 2:**
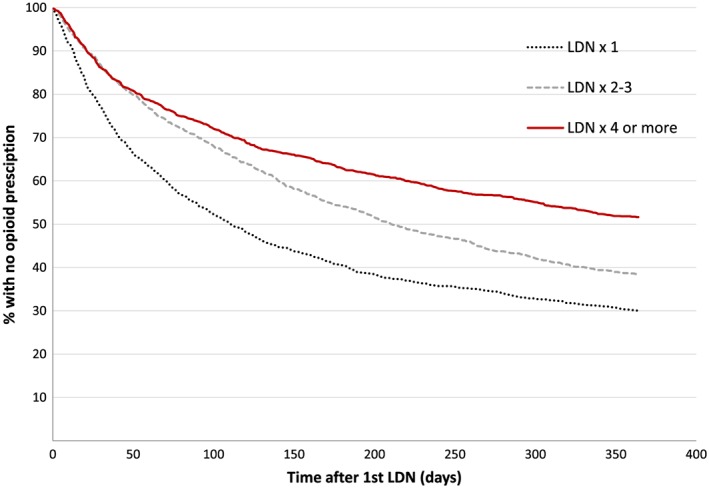
Time to first opioid prescription after starting LDN. The proportion of patients that had not collected an opioid prescription by time since the first LDN prescription. Data presented in three patient cohorts defined by number of LDN prescriptions collected in the study period. [Colour figure can be viewed at wileyonlinelibrary.com]

The proportion of patients within each subgroup that had not collected an opioid prescription since the first LDN prescription is plotted by time in Figure 2. Time to first opioid prescription increased by the number of LDN prescriptions collected. In the LDN × 1 group, 30.0% did not collect opioids at all, against 51.6% of the LDN ≥4 group. Fifty percent of the LDN × 1 group had collected a prescription on an opioid within 111 days, compared with 211 days among patients in the LDN × 2–3 group.

**Figure 3:**
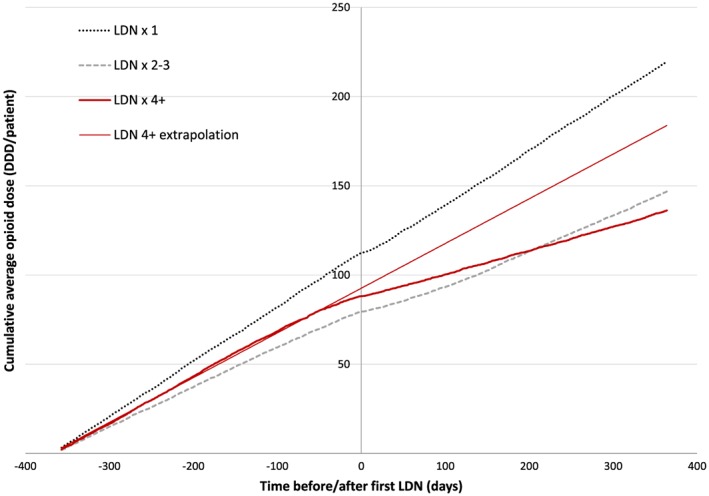
The effect of LDN on opioid consumption. Cumulative average opioid dose according to NorPD 1 year before and after the first LDN prescription in subgroups defined by number of LDN prescriptions collected. As a visual aid, an extrapolated linear regression line of opioid consumption 1 year before the first LDN prescription in the LDN ≥4 subgroup is added. [Colour figure can be viewed at wileyonlinelibrary.com]

Figure 3 shows the average cumulative opioid dose in the three subgroups before and after the first LDN prescription. The slope and absolute values of the curves show that collection of LDN prescriptions was followed by an immediate and lasting reduction in opioid consumption in the LDN ≥4 group. In the LDN × 1 group, there was minimal change in number of DDDs collected on opioids. The LDN × 2–3 group also reduced the average number of DDDs on opioids collected, but the association was less pronounced and temporary compared with the LDN ≥4 group.

**Figure 4:**
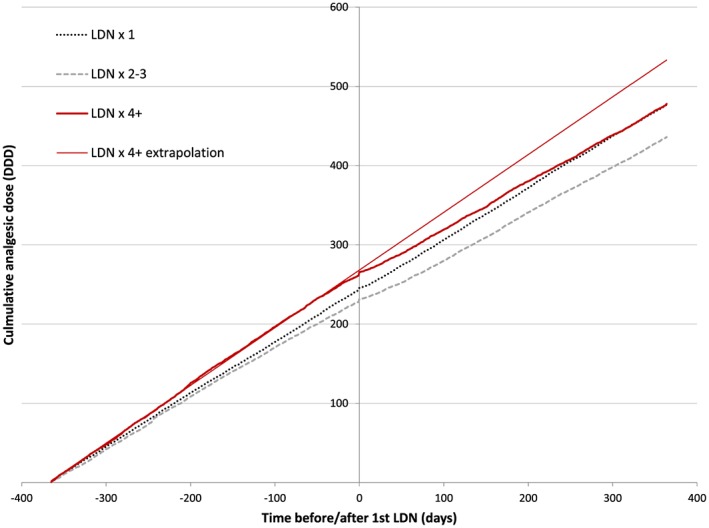
The effect of LDN on total analgesic consumption. Cumulative average dose of opioids, NSAIDs, other analgesics and antipyretics according to NorPD 1 year before and after the first LDN prescription in subgroups defined by number of LDN prescriptions collected. As a visual aid, an extrapolated linear regression line of opioid consumption 1 year before the first LDN prescription in the LDN ≥4 group is added. [Colour figure can be viewed at wileyonlinelibrary.com]

As can be seen from Figure 4, initiation of LDN was followed by reduced consumption of the total analgesic use (opioids, NSAIDs, other analgesics and antipyretics combined) in patients that collected LDN more than once. After LDN, patients in the LDN ≥4 group went from the highest total consumption of prescription analgesics to a level similar to the LDN × 2–3 group.

## Discussion

### Key results

The Norwegian “LDN tsunami” is a unique natural experiment that enables examination of possible associations to prescribing of other drug classes in large groups of patients. Collection of prescriptions on LDN was associated with collection of a reduced number of DDDs on opioids in the first year after the first LDN prescription. There was a dose response effect where increasing number of LDN prescriptions was associated with increasing reduction in number of DDDs of opioids collected. We observed no increase in the number of DDDs collected on NSAIDs or other analgesics and antipyretics corresponding to the decrease in number of DDDs on opioids.

### Limitations

There are important limitations to this study. Because of the massive demand after the TV documentary, the domestic LDN product went from being a narrow small‐scale pharmacy producing drug to a high‐volume product in the standard pharmacy assortment. Until receiving a national product identification number on 15 May 2013, LDN prescriptions were not captured by NorPD. Consequently, a number of individuals in the LDN × 1 group and LDN × 2–3 group are possibly assigned to a subgroup with too few LDN dispenses. The effect of this possible exposure bias is difficult to predict. However, the observed dose response association between the number of LDN prescriptions and reduction in number of DDDs on opioids suggests a limited effect. Other sources of LDN not captured by NorPD were, for example, dilution of full‐dose naltrexone tablets in water and naltrexone from foreign sources. This was negligible because only 77 persons collected at least one prescription of full‐dose naltrexone (50 mg) in Norway in 2013 according to NorPD data available to us.

Age and gender distribution was similar in the three LDN subgroups, but there were important differences in the consumption of analgesics. The patients in the LDN × 1 group collected almost 25% more opioids before starting LDN than the LDN ≥4 group, and the LDN ≥4 group had the highest total consumption of all analgesics combined. This probably reflects different morbidity in the subgroups. Possibly, there was a higher proportion of opioid‐dependent patients in the LDN × 1 group preventing further LDN use. The higher use of other analgesics and antipyretics and NSAIDs in the LDN ≥4 group may explain a lower need for opioids. In this subgroup, the high total analgesic use (opioid, NSAIDs, other analgesics and antipyretics combined) may suggest more severe diseases or more adequate pain therapies.

All opioids require prescription in Norway, and we are confident that we have almost complete data on collected prescriptions on opioids among the included. OTC is not captured by NorPD, and the number of DDDs on NSAIDs and other analgesics and antipyretics is higher than reported in this study. However, it is likely that a majority of the patients have chronic diseases that qualify for reimbursement of other analgesics and antipyretics and NSAIDs, and therefore buy OTC to a lesser extent than the general population. From 2013 to 2014, the annual sale of paracetamol in Norway was almost unchanged, but the proportion sold OTC was reduced by 3%.^18^ However, a recent study found that the prevalence of OTC analgesic use was highest in patients with chronic pain and intermittent opioid use.^19^

The collection of at least one opioid prescription before starting LDN was an inclusion criterion in this study. It could be argued that a reduction in opioid consumption is as expected, representing regression to a mean. However, the observed dose response effect suggests additional independent association with LDN. Repeated collection of LDN was associated with a more pronounced reduction in opioid use and longer time to first opioid prescription.

The consumption of opioids per person was 13 times higher among the included patients than the average for the entire population.^20^ Among all non‐cancer opioid users in the Norwegian population, mean opioid consumption in 2007 was 59 DDDs per person per year,^21^ which is lower than the mean opioid number of DDDs before starting LDN in this study. Interestingly, the number of DDDs on opioids in the LDN ≥4 group fell to an even lower level (49 DDDs per person per year). Change of therapy or combination use of more potent opioids may reduce the number of DDDs. *Post hoc* analysis showed that in the LDN ≥4 group, there was a reduction in number of patients using strong opioids, while the number increased in the other subgroups (data not shown).

We only included patients who collected a prescription on opioids during 1 year before starting LDN. In a *post hoc* analyses, we have identified 1183 additional persons who collected opioids only after the first LDN prescription. Among these, 59% collected opioid only once compared with 22% among those included.

Some other drug classes have a place in the treatment of pain but are not included in this study. Theoretically, some patients may have compensated less use of opioids with increased use of antidepressants and antiepileptics. However, the main indications for these drugs make it reasonable to exclude them from our analyses.

### Interpretation

The observed changes in opioid use were both statistically and clinically relevant. The dose response effect of LDN exposure suggests an association between initiation of LDN therapy and the reduction of opioid consumption. In contrast, the average opioid use per person in Norway increased by 1.7% from 2013 to 2014.^22^ However, our data do not provide any evidence on causality of the mechanisms behind the findings. A probable explanation is that the patients were aware of the precautions on concomitant use and therefore avoided opioids while taking LDN. In light of the numerous warnings against LDN–opioid combinations, the opioid consumption in the LDN ≥4 group is probably considered surprisingly high by some Norwegian LDN advocates. The minimal association on number of DDDs of opioids collected among the LDN × 1 group may be related to withdrawal symptoms or need for opioids. In the LDN × 2–3 group, many probably terminated LDN therapy during the study period. As seen from Figure 2, the proportion of opioid users in this cohort went from initially being similar to the LDN ≥4 group to become more like the LDN × 1 group. Individuals not belonging to the LDN ≥4 group may have preferred to continue on opioids rather than LDN.

The scientific basis for discouraging the combination of LDN and opioids is weak. In opioid‐dependent patients, it may be relevant. There are reports on naltrexone‐induced withdrawal syndrome in opioid‐dependent patients.^23^ On the other hand, it is suggested that opioid antagonists in low doses are useful in the treatment of opioid withdrawal.^24^ Data also suggest that naltrexone in low doses could be used to prevent intolerable morphine adverse events^25^ or even inhibit the development of opioid tolerance.^26^

A more controversial explanation to the reduction in number of DDDs on opioids is that the patients experienced therapeutic effects against their medical condition. After all, this is their main motivation for trying LDN. This is partly supported by the observation that the large reduction in number of DDDs on opioid was not fully compensated by an increase in NSAIDs or other analgesics and antipyretics. In fact, the number of DDDs of NSAIDs was reduced in all subgroups, while the use of NSAIDs and other analgesics and antipyretics in Norway increased by 3.1% during the same period.^22^

Norwegian Prescription Database contains very limited clinical information, and we were unable to observe any changes in pain symptoms. It is also possible that there was a selection of patients with a higher pain threshold to the LDN ≥4 group, meaning that these patients accepted more pain to be able to continue LDN. Two small preliminary studies have shown some beneficial effects on fibromyalgia,^7^, ^8^ and we cannot rule out that such effects are reflected in our findings. It is important to emphasize that this study includes all patients that collected LDN in 2013. We are therefore not able to estimate how much of the observed decline in the use of painkillers was attributable to patients with chronic pain or to patients with other conditions.

### Generalizability

This is the first study based on data from pseudonymized patients on the association of LDN prescribing and opioid consumption. It is a registry‐based study covering the entire Norwegian population, which means that our results reflect the actual domestic situation. However, the popularity of LDN in Norway has until now been unparalleled, and it is an open question whether prescribing of LDN will affect opioid users similarly if a “LDN tsunami” should strike other countries. To what extent LDN users are warned against concomitant opioid use will be crucial. If LDN use has therapeutic effects that reduce the need for opioids, it is likely that LDN use will lead to similar changes in opioid use elsewhere. It would be interesting to examine whether prescribing LDN for chronic pain can be an effective measure against overuse of prescription opioids in this vulnerable patient group. The heterogeneity of our study population is enormous with a multitude of different indications for LDN use. Therefore, randomized, double blind, clinical effect studies on LDN in pain conditions are needed.

## Conflict of Interest

Both authors have completed the Conflict of interest disclosure form (available on request from the corresponding author) and declare no support from any organization for the submitted work.

**Key Points**

- Low‐dose naltrexone (LDN) use was followed by reduction in opioid use in patients that collected LDN more than once
- There was a dose–response relationship between increasing LDN exposure and reduction in total opioid use and increasing time to first opioid prescription after starting LDN
- Initiation of LDN was followed by a 46% annual reduction opioid prescriptions collected among persistent LDN users
- The reduction of opioid use was not compensated by increased use of other prescribed painkillers

## Ethics Statement

The project protocol was submitted to the Regional Committee for Medical and Health Research Ethics of North Norway. The committee concluded that disclosure was not mandatory because of pseudonymized data. The local privacy ombudsman for research at the University Hospital of North Norway approved the project (#0449). For Norwegian central health registries, like NorPD, consent from individual patients is by law not required.

## Author Contributions

G. R. contributed to the conception of the study, designed the study, performed statistical analyses and drafted the manuscript. L. S. contributed to the design of the study and to statistical analyses. Both authors participated in revising the manuscript and approving the final version.
